# Virtual Reality for Research in Social Neuroscience

**DOI:** 10.3390/brainsci7040042

**Published:** 2017-04-16

**Authors:** Thomas D. Parsons, Andrea Gaggioli, Giuseppe Riva

**Affiliations:** 1 Department of Psychology, University of North Texas, Denton 76203, USA; 2 Computational Neuropsychology and Simulation (CNS) Laboratory, University of North Texas, Denton 76203, USA; 3 Department of Psychology, Università Cattolica del Sacro Cuore, Milan 20123, Italy; andrea.gaggioli@unicatt.it (A.G.); giuseppe.riva@unicatt.it (G.R.); 4 Applied Technology for Neuro-Psychology Laboratory, Istituto Auxologico Italiano, Milan 20145, Italy

**Keywords:** social neuroscience, ecological validity, virtual reality, virtual characters, social presence

## Abstract

The emergence of social neuroscience has significantly advanced our understanding of the relationship that exists between social processes and their neurobiological underpinnings. Social neuroscience research often involves the use of simple and static stimuli lacking many of the potentially important aspects of real world activities and social interactions. Whilst this research has merit, there is a growing interest in the presentation of dynamic stimuli in a manner that allows researchers to assess the integrative processes carried out by perceivers over time. Herein, we discuss the potential of virtual reality for enhancing ecological validity while maintaining experimental control in social neuroscience research. Virtual reality is a technology that allows for the creation of fully interactive, three-dimensional computerized models of social situations that can be fully controlled by the experimenter. Furthermore, the introduction of interactive virtual characters—either driven by a human or by a computer—allows the researcher to test, in a systematic and independent manner, the effects of various social cues. We first introduce key technical features and concepts related to virtual reality. Next, we discuss the potential of this technology for enhancing social neuroscience protocols, drawing on illustrative experiments from the literature.

## 1. Introduction

Social neuroscience is an interdisciplinary field that investigates how social processes and behaviors are implemented by biological systems [1,2,3]. A specific focus of social neuroscience is on the paradigm of social cognition, intended as the “the study of mental processes involved in perceiving, attending to, remembering, thinking about, and making sense of the people in our social world” [4]. Research in this domain investigates the neural and neuro-physiological correlates of cognitive processes found in a wide spectrum of social interactions. This goal is achieved through a broad range of methods, which include recordings of intrinsic electrical activity of the brain, brain stimulation, neuroimaging, psychophysiological measurements, biomarkers, and computational modeling. These methods are either used alone or in combination with more traditional research tools, such as behavioral observation and self-report methods.

Many of the pioneering paradigms in social neuroscience reflect a noteworthy emphasis upon laboratory control and experiments that involve participants observing static stimuli (e.g., simple, static representations of socially relevant stimuli; static photographs of emotionally valenced facial expressions). In addition to studies using the International Affective Picture System [5] or standard facial expressions [6], social neuroscientists have used sentences [7,8], paragraphs [9,10] and scripted imagery of social events [11]. Unfortunately, static and simplistic stimuli may not be representative of real-life social encounters, and tasks with static stimuli may result in ceiling effects [12,13]. Moreover, static stimuli tend to be non-natural due to target processes (viewing a facial expression) not occurring as isolated events in real life. Instead, they are closely related to cues and contextual data. According to Zaki and Ochsner [14], real life social exchanges are multimodal (visual, semantic, and prosodic information), dynamic, and contextually embedded so that participants are presented with environmental information that has framed their interpretation of another’s internal states. Some researchers have used video clips to present dynamic social stimuli in a controlled setting [15]. Although this allows participants to passively observe the social interactions of others, it does not allow the participants to actively experience the social interaction. Given these concerns, several researchers are beginning to question whether the knowledge gained using such stimuli will have general applications for the social cognitions involved in everyday activities [16,17,18,19,20].

Virtual environments offer a methodology for presenting digitally recreated simulations of real-world activities. Recent advances in the software and hardware platforms used in virtual environments, allow for the presentation of dynamic social stimuli and emotionally engaging background narratives [21,22]. These new virtual environment platforms may engender a sense of presence in users that can serve to enhance the representativeness and generalizability of findings from the virtual world to real world social cognitions [23,24]. In this review, we aim to describe the potential of virtual environments for the presentation of dynamic social stimuli that can be used for assessment of social cognitions while participants are immersed in simulations of real world social contexts. Several studies using virtual environments to explore social cognitive processes are discussed. Although social neuroscientific research is emphasized throughout, a number of examples provided reflect assessments of behavioral performance. While not exhaustive, this review surveys research that highlights the ways in which participants respond to social stimuli in simulations that approximate real-world activities and interactions.

## 2. Virtual Reality and Virtual Environments: Key Definitions

The term Virtual reality (VR) refers to a computer-generated environment in which the user can perceive, feel and interact in a manner that is similar to a physical place. This is achieved by combining stimulation over multiple sensory channels—such as sight, sound and touch—with force-feedback, motion tracking, and control devices. In an ideal VR system, the user would not be able to distinguish an artificial environment from its physical counterpart. Whilst none of the current VR systems would be able to pass this criterion, significant advances in the perceptual fidelity of virtual environments have been achieved over the last few years.

### 2.1. Components of a Virtual Reality System

From a technological viewpoint, the essential components of a VR system are input devices, output devices, and the simulated scenario (i.e., the virtual environment). Input devices allow the user to interact with the virtual world, by capturing the user’s actions (e.g., head, limb, and hand movements) and sending this information to the computer. Virtual reality platforms also include various tracking devices (e.g., data gloves, head-positioning sensors, eye-tracking), pointing devices (e.g., six-degrees-of-freedom mouse, trackball, joystick etc.), and audio devices (surround sound, audio recording, speech recognition). Output devices convey continuous, computer-generated information to the user through multiple sensory modalities: visual, auditory, olfactory, and haptic (tactile) feedback. For most VR applications, however, sight is still the most important sensory modality, which is usually implemented through stationary displays (such as such as projection and fish-tank) or head-mounted displays. Finally, the virtual environment is a computer-generated three-dimensional model of a physical environment, in which the user can navigate and interact with the objects that it contains. These virtual objects are linked by specific behaviors and dynamic properties, which also specify the full range of manipulations that the user can perform with them (e.g., picking, pushing, rotating, resizing etc.). In multi-user virtual environments (MUVEs), two or more users are co-located in the same virtual place and have the possibility for communicating and collaborating using *avatars*, which are personalized, graphical representations of the self within the virtual world that are directly controlled by the user, in real time. However, virtual environments may include other social entities that are not acted on by human beings, but are driven by the computer itself by means of an artificial intelligence program (an *embodied virtual agent*) [25,26].

### 2.2. Immersion and Presence in Virtual Environments

VR systems are usually classified as immersive or non-immersive [27]. Although a precise definition of immersion remains elusive, the current working notion is that an immersive system should be able to sensorily isolate the user from the physical world, and replace the sensory streams coming from the real world with rich synthetic stimuli generated by the computer [28]. The type of VR system that best fits this definition is the Cave Automatic Virtual Environment (CAVE) [29]—a room-sized cube which surrounds a user with rear-projected screens (including floor and ceiling) on which images can be depicted in stereoscopic modality. Head-mounted displays (HMDs) fall also in the category of immersive systems, since they completely occlude any visual contact with the outside world and replace it with computer-generated images, which are dynamically adapted to any viewing position by means of head-tracking. Non-immersive VR systems—sometimes also described as a “Window on a World”—typically mimic a virtual environment using a standard high-resolution monitor, and feature limited interactive capabilities (e.g., no head-motion tracking). Examples of non-immersive VR include most 3D-videogames and desktop-based 3D modelling applications. Whereas immersion refers to the objective degree to which a VR system is able to surround the user with vivid and interactive multi-sensory stimulations [30], the concept of presence is used to describe the subjective feeling of “being there” within a virtual environment. The definition of presence was introduced by Sheridan [31], who described it as “the effect felt when controlling real world objects remotely” (pp. 123–124). Starting from this definition, several theoretical accounts of presence have been developed, which can be included into two main perspectives.

The first conceptualization sees presence as an experience resulting from the interaction of a user with a given medium. According to this perspective, presence is a psychological state that is shaped, although not completely determined by, the immersive properties of the medium [32]. For example, a fully-immersive system such as a CAVE may evoke more presence than a desktop-based virtual environment; however, individual differences among users could also play a role [33,34].

The second perspective (which will be more extensively discussed in Section 3) does not conceptualize presence as a function of a given medium, but sees it as an embodied phenomenon, which is linked to the organization and control of action. According to Zahorik and Jenison [35], presence is “tantamount to successfully supported action in the environment” (pp. 79–80). In this definition, the sense of “being there” is strictly related to the subject’s ability to successfully “act there”, in the space where he/she is situated—either “physical” or “virtual”. Central to this conceptualization of presence is Gibson’s notion of affordance, intended as clues in the environment that indicate possibilities for action [36]. In the specific case of VR, the more effectively the virtual environment allows the user to successfully engage in an intended behavior, the more present he/she will feel.

A further distinction can be made between presence and social presence. The concept of social presence was originally developed by Short et al. [37] to describe the degree of salience between two communicators using a communication medium. When applied to VR, the social presence construct is used to understand how users perceive the presence of other social entities (living or synthetic) in a virtual environment.

These various theoretical stances on presence/social presence have led to different approaches for operationalizing and measuring these constructs. The most common methodology is based on the use of self-report questionnaires, which are administered to the participant after he/she has been exposed to the virtual environment. However, these subjective measures have been criticized by some scholars, since post-test assessments of the user’s feelings of presence are dependent on memory of the event, and thus prone to recall errors. To overcome the limitations of retrospective assessments of presence, more “objective” measurement methods have been introduced that involve monitoring behavioral or psychophysiological responses during the virtual experience [38] and there is a growing emphasis upon physiological and behavioral assessment, as well as the relation between immersion and affective responding [39,40]. Furthermore, neuroscience techniques are being more commonly used for presence measurement, such as functional magnetic resonance imaging (fMRI) [41], transcranial Doppler [42], and electroencephalography [40].

## 3. Being Present in Virtual Reality: From the Self to the Others

### 3.1. Feeling Present in Virtual Reality

The user experience in virtual reality is significantly affected by the extent to which users experience themselves as being present in the mediated world that the VR makes available to them [43,44]. This feeling of “presence”, which can be operationally defined as “the perceptual illusion of non-mediation” [45], is a crucial aspect of many recent and developing interactive technologies [44,46]. When users experience it, they feel that the technology has become part of their bodies and that that they are experiencing the virtual world in which they are immersed. Moreover, when they feel present in VR, they react emotionally and bodily (at least to some extent), as if the virtual world exists physically. However, as Biocca [47] pointed out, “while the design of virtual reality technology has brought the theoretical issue of presence to the fore, few theorists argue that the experience of presence suddenly emerged with the arrival of virtual reality”. In this view, we suggest that, as the natural, unmediated sense of presence is grounded in the perceptions, actions and experiences of “being” in a body [48,49], so thus the experience of presence in a technologically-mediated environment is to some extent a function of the possibilities for interaction, including social interaction [50,51]. Specifically, we suggest that the sense of presence, the feeling of being in a body and in the space around it, could be the output of an evolved neural representation, or neurotag [52], “a large groups of brain cells that are distributed across multiple brain areas and that are thought to evoke a given output”, (p. 1). This allows for the solving of a key problem for survival: how to differentiate between the internal and the external [53,54,55,56]. In humans, this skill has developed into two different competences.

#### 3.1.1. Presence: Inside the Body vs. Outside the Body

The first competence is the ability to distinguish between internally generated intentions and/or beliefs, and externally enacted actions (presence: inside the body vs outside the body). This distinction cannot be the sole result of emotional appraisal or reality judgments [46], because imagined situations trigger the same emotional responses as physical situations [57], and both imagined and physical events may also be judged as either real or unreal. Various authors [58,59,60,61] have suggested that this ability is based on stochastic models of the environment, which are updated on the basis of processed sensory information: the brain, along with the actual motor action, instantiates a sophisticated simulation of its sensory outcome (predictive coding), based on motor codes, and uses this to monitor any possible variation in its course.

#### 3.1.2. Social Presence: Self versus Others

The second competence is the ability to distinguish between internally generated actions from actions generated from other humans (Social Presence: Self versus Others). The importance of this distinction is underlined by the recent discovery of “mirror neurons” (F5c-PF) that are activated both when the subject performs an action, and when the subject sees another individual performing the same action [62,63]. An explanation of the dual activation of these neurons arrives from the Common Coding Theory: according to this theory, motor representations (actions to be performed) and perceptual representations (actions perceived) are coded by our brain using the same motor code [64,65]. In practice, during all the phases of an action—*planning* (“I want to move my hand to pick up an object”), *execution* (“I move my hand and pick up the object”) and *interpretation* (“I see another person move their hand to pick up the object”), the subject is activating the same areas of the brain. Moreover, it suggests that, at the neural level, the action performed and the action observed are codified in a multi-subjective format that does not allow a direct distinction between the actor and the observer. As Becchio and Bertone point out [66]: “By codifying an agent-free representation of action, mirror neurons support the visual and motor comprehension of the action, but are not in themselves enough to attribute an action to an agent. This level of comprehension, defined as agentive by the authors, requires that the agent parameter is specified as a separate parameter: only in this way does the action become the action of a particular agent” (p. 859).

### 3.2. The Layers of Social Presence

Cognitive science is now describing actions as the result of a complex intentional chain that cannot be analyzed at a single level [67]. Pacherie [68] identifies three different “levels” or “forms” of intentions, characterized by different roles and contents: distal intentions (D-intentions), proximal intentions (P-intentions) and motor intentions (M-intentions):

- *D-intentions (Future-directed intentions):* These high-level intentions act both as intra- and interpersonal coordinators, and as prompters of practical reasoning about means and plans. A possible D-Intention is the intention to become a doctor. This intention hides a complex personal path involving many interpersonal relations and achievements that the subject is not fully able to control and predict in advance: the individual should obtain a Bachelor’s degree first, then attend medical school and take part in rotations, residencies, and exams. Then the individual should choose a specialty and train for it. Finally, the individual should apply for a residency program, training, and certification.
- *P-intentions (Present-directed intentions)*: These intentions are responsible for high-level (conscious) forms of guidance and monitoring. They have to ensure that the imagined actions become current through situational control of their unfolding. Within the above D-intention—to become a doctor—an example of P-intention is to join the Anatomy class: the individual should leave home, take a car or a bus to reach the university, finding the right building and entering the class during the scheduled time. Differently from before, the individual is able to predict in advance all the different steps required to enact the intention. In this way, they can use it to control its unfolding, and find situational solutions to possible problems. For example, if the car does not start, the individual can use the bus to reach the university.
- *M-intentions (Motor intentions)*: These intentions are responsible for low-level (unconscious) forms of guidance and monitoring: we may not be aware of them, and we have only partial access to their content. Further, their contents are not propositional. An example of M-Intention within the above P-intention—to join the Anatomy class—is moving the steering wheel while driving the car. This intention, being subpersonal and unconscious (the individual is not aware of the specific movements done during driving) allows for the monitoring and control of the body movements, and the interaction of the body with the surrounding environment.

In this view, if Social Presence permits the identification of the Other’s intentions through the analysis of the Other’s actions, it should be able to differentiate between these intentions. Putting together the development of imitative skills with the structure of the intentional chain, it is possible to suggest that Social Presence includes three different layers/subprocesses that are phylogenetically different, but mutually inclusive [51,69,70]:

- Other’s Presence (Other vs. the Self—M-Intentions)
- Interactive Presence (Other toward the Self—P-Intentions)
- Shared Presence (Other is like the Self—D-Intentions)

The first layer of Social Presence is the “*Other’s Presence*”—the ability to recognize motor intentions, which allows the Self to recognize and imitate an intentional Other. The better the subject is able to *recognize within the sensorial flow a motor intention produced by “an Other similar to the Self”*, the better the subject is able to carry out an intention, and thus increase the subject’s chances of survival (the Other in opposition to the Self).

The second level of Social Presence is “*Interactive Presence*”, the ability to recognize motor and proximal intentions. This ability allows the subject to identify the Other whose intention is directed towards him. The better the subject is able to *recognize within the sensorial flow, the motor and/or proximal intentions direct towards him/her by “an Other similar to the Self”*, the greater the chances of successfully carrying out a social action, and therefore the greater the chances of survival (the Other towards the Self).

The third level of Social Presence is “*Shared Presence*”, which is the ability to recognize motor, proximal and distal intentions. This ability allows the subject to identify another whose intentions correspond to the subject’s own. The better the subject is able to *recognize within the sensorial flow an “Other similar to the Self”, with intentions the same as the subject’s own*, the better the subject will be able to successfully initiate collective intentions—which call for a form of cooperation that is not a mere coordination between subjects; rather, it involves mutual understanding of the intentions of the other subjects [71]—increasing the subject’s chances of survival (the Other like the Self). These three levels are evolutionarily organized from the lowest to the highest. However, the levels of social presence are not functionally separate, but mutually inclusive. This leads to two consequences. The superior levels also include the inferior levels: if the subject is able to understand distal intentions (shared presence), the subject is also capable of understanding motor intentions (other’s presence). At the same time, it is impossible to activate the higher levels of social presence if the lower levels are not activated first. If one unable to understand a subject’s proximal intentions (interactive presence) then one will not be able to understand the subject’s distal intentions (shared presence).

The three levels of social presence are linked by *simultaneous influences on the subject’s capacity for social interaction*. The way in which the interaction is experienced, changes depending on the level of social presence experienced by the subject. It is important to note that the subject is unaware of the role of social presence in determining the characteristics of the subject’s actions. The subject is, however, evolutionarily programmed to perceive the shift from one level of social presence to another, within social interactions. As underlined by Waterworth and Riva: “if this shift offers a valuable opportunity, the subject can act to increase his level of social presence. For example, if a girl starts staring at me at a party, I immediately become aware of the shift from other’s presence (the girl is at the same party as me) to interactive presence (the girl is looking at me). If the girl is interesting, I can approach her and talk to her in order to understand her intentions. Is she looking at me because she likes me or because I have a stain on my jacket?” [46] (p. 112).

## 4. Enhancing Social Neuroscience Protocols with Virtual Environments

Although the use of VR in social neuroscience is still in its infancy with respect to more established methodologies (psychophysiology, neuroimaging, brain stimulation), there are several advantages that VR can offer to this field. The first, and perhaps most obvious asset offered by this technology, is that it allows the generation of life-like simulations of social information. As noted by [15], the stimuli that are used in many social neuroscience studies lack important aspects of actual live social interaction; this concern is shared by other authors [72,73,74,75]. On the other hand, the use of unnaturalistic stimuli is necessary in order to exert control on potential extraneous variables, which would be difficult or impossible to control in field or natural experiments. As the following examples illustrate, VR may represent the optimal tradeoff between ecological validity and need for experimental control of outside influences, since it can be used to generate stimuli that approximate the complexity of a social situation in the well-monitored environment of the laboratory [22,76,77].

### 4.1. Virtual Reality for Multimodal, Dynamic and Contextually-Rich Stimulus Presentation

Zaki and Ochsner [14] have emphasized three key differences between the stimuli used in social neuroscience experiments and real-life social situations. First, laboratory stimuli are usually unimodal (that is, presented through a single channel), whereas “naturalistic” social cues are multimodal, involving visual, semantic, and prosodic information. Second, the stimuli lack the dynamic properties of real-world social phenomena (changing over time). Third, research stimuli often represent isolated “snapshots” of the social information stream, whereas in real-life, social cues are contextually embedded so that their interpretation is constrained by the processing of concomitant or antecedent social information.

Virtual reality allows for the presentation of social information in a multimodal approach that integrates the auditory, visual and even haptic perception of social cues. Furthermore, VR enables the experimenter to present dynamically-adaptive 3D stimuli, that is, stimuli that can change their phenomenical properties (e.g., shape, intensity, position, etc.) in real time according to specific interaction rules defined by the researcher. In this way, VR can be used to generate arbitrarily-complex chains of events which approximate the degrees of freedom that characterize real-life social interactions. Finally, VR can embed the stimuli in a rich context of social information that helps increase the participant’s feelings of social presence and acceptance of an agent as an intentionally engaging social entity [78].

### 4.2. Virtual Reality Allows for Automated Multimodal Data Logging and Analysis

Virtual reality provides the option of automatically-logging participants’ behavior, responses and performance during social neuroscience experiments. The tracking systems and the other input devices that are used to operate a VR system represent, at the same time, an excellent means for recording the user’s actions in the virtual environment in an accurate and quantifiable manner. These tools can be used, for example, to precisely track users’ body movements, gestures, and posture, and for collecting verbal signals. This opportunity is enabled by the availability of powerful interface tools such as the popular Virtual-Reality Peripheral Network (VRPN), which is a device-independent and network-transparent system for accessing virtual reality peripherals in VR applications [79].

Furthermore, the use of VR allows for recording not only information sources in real time, but also allows the integration of data captured by different tracking devices, each monitoring a separate behavior channel. The potentialities of this approach have been explored by Steptoe and Steed [80], who have designed a reference architecture that allows for multimodal data capture and analysis in immersive collaborative virtual environments. The logging architecture proposed by these authors is able to collate multiple components of a user’s nonverbal and verbal behavior in a single log file, in a way that preserves temporal characteristics, and emphasizes the causal interrelationships between synchronous tracking streams.

### 4.3. Virtual Reality for Control and Flexibility in the Administration of Social Cues

A further methodological benefit of VR is represented by the high degrees of control and flexibility that the experimenter has when defining and administering the stimulus [21,22]. VR enables the researcher to selectively manipulate variables that in naturalistic situations cannot be independently investigated. For example, a methodological issue in the study of non-verbal communication is that implicit cues such as facial expressions, gestures, eye contact, and posture, are generated outside of the individual’s awareness, and are inextricably intertwined with verbal behaviors, making it difficult to test their effects separately. The use of anthropomorphic virtual characters (VCs) may help to overcome this challenge. VCs enable the experimenter to control body motion independently from body shape, allowing the decoupling of top-down effects of representation from bottom-up effects of behavior [81,82]. Bailenson et al. [83,84] have exploited this methodological advantage by introducing the “Transformed Social Interaction” (TSI) paradigm. This approach aims at using avatars to systematically investigate the role of nonverbal signals (e.g., eye gaze, facial gestures, and body gestures) in interpersonal communication, by artificially enhancing or degrading the signals. Implicit cues are captured using motion tracking techniques and then rendered through a virtual representation of the interacting members. By filtering and modifying nonverbal behaviors, the TSI approach aims at identifying how implicit cues shape the characteristics and the course of social interactions.

A growing body of empirical evidence supports the validity of VCs as stimulus material in social neuroscience. Both behavioral and neuroimaging studies have consistently shown that participants’ perception of human-like avatars does not significantly differ from the perception of real human beings, triggering comparable behavioral, physiological and neuronal activations [85]. This perceived similarity is not limited to the visual appearance of virtual characters, but also extends to the recognition of emotionally-linked facial and bodily expressions [86,87,88].

## 5. Virtual Reality for Simulating Impossible Social Interactions

Not only can social neuroscientists use VR to reproduce real social scenarios, but also to create “impossible stimuli”, that is, situations that break the physical laws of nature. For example, Friedman et al. [89] described a method based on immersive virtual reality for generating an illusion of having traveled backward through time to relive a sequence of events in which the individual can intervene and change the course of history. Participants were involved in a moral dilemma in which they experienced a sequence of events (in this case, the opportunity to stop a gunman from killing others) in VR from a first-person perspective. In the time travel condition, after participants traveled back to the present, they assessed their past selves with the potential for changing events. This manipulation resulted in an increase in participants’ guilty feelings about the events that had occurred, and an increase in the support of utilitarian behavior as the solution to the moral dilemma.

### 5.1. Virtual Representations of Self and Other

Immersive virtual environments use flexible computer platforms that allow the researcher to manipulate the type, form, shape, and size of virtual human bodies. Studies of social cognition that emphasize shared bodily representations for Self and Other can be performed to assess participant perceptions, attitudes, and behaviors [90]. An example is the use of VR to simulate impossible real-world social interactions or embodying the participant in a virtual body whose appearance (size, anatomy, race, gender, age, etc.) can be manipulated [77,91,92]. An example of this approach is the “Proteus effect” [93], in which a user’s avatar alters specific characteristics of the physical self. A series of studies that have applied this paradigm have shown that when the user interacts with another person via the avatar, his/her behavior is shaped by the type of representation in the virtual world [93,94].

Studies have explored the social behaviors of participants exposed to virtual humans from other demographic groups [95,96]. Moreover, interactive virtual humans have been used to observe the influence of virtual embodiment on implicit social cognitions related to several areas: race [97,98]; gender groups [99]; age [100]; and body shape/size [101,102,103]. Peck and colleagues (2013) [98] demonstrated that embodiment of light-skinned participants in a dark-skinned virtual body significantly reduced implicit racial bias against dark-skinned people, in contrast to embodiment in light-skinned, purple-skinned or no virtual body. Another example is Banakou and colleagues [100] immersion of adult participants in virtual child bodies to examine the association between implicit attitudes and embodiment. The adults inhabiting the virtual child's body overestimated the size of objects and demonstrated what appeared to be child-like implicit attitude and behavioral changes. When participants were immersed in an adult body that was the same size as that of a child, the adult participants did not exhibit these changes. Furthermore, embodying adult males in a female child virtual body has been found to produce strong physiological responses when the child is placed in a threatening situation [99].

### 5.2. Virtual Characters for Interpreting Others

Virtual characters have been used for investigations of brain regions involved in interpreting others’ face and eye movements. When approached by a virtual character exhibiting an angry expression, increased activation was found in the participant’s superior temporal sulcus, lateral fusiform gyrus, and a region of the middle temporal gyrus [104]. Studies have also used virtual characters for studies of joint attention [18,105,106,107,108,109]. Schilbach and colleagues [18,108] have introduced dynamic virtual characters in the scanner to characterize the neural correlates of being involved in social interactions. In similar research, Wilms and colleagues [109] used the eyetracking data of participants lying inside a scanner for real-time control of a virtual character’s gaze behavior that was responsive to the participant’s gaze. This allowed investigation of neural differences between joint attention and simple gaze following. Results revealed a main effect for joint attention and activation of the medial prefrontal cortex, posterior cingulate cortex, and the anterior temporal poles. For each of these studies, joint attention-related activity was found in brain regions that have been associated with social cognition: the medial pre-frontal cortex [105,107,108], posterior superior temporal sulcus [105,107], temporoparietal junction [105,107], and the occipital gyrus [106], as well as the insula, precuneus, and amygdala [105].

## 6. Virtual Reality-Based Moral Dilemmas

### 6.1. Virtual Trolley Dilemma

An example of moral decision making can be found in the text-based “trolley problem” including both the “switch” and “footbridge” dilemmas [110]. During the switch dilemma, the participant must choose whether to agree with switching a trolley’s direction to slay one worker instead of five. Typically, the majority of participants approve [111]. For the footbridge dilemma, the participant is presented with the dilemma of pushing a bulky man from a bridge to halt an approaching trolley that would otherwise slay five workers. Most participants report disapproval of this pushing the man off the bridge. Dual-process frameworks have been used to explain the different participant responses to the switch and footbridge dilemmas [111,112]. In the dual process approach of Greene and colleagues [111] automatic emotional processing is related to non-utilitarian (i.e., deontological) judgments, and controlled cognitive processing is related to utilitarian judgments that maximize welfare for the largest number. Per this model, participant responses are impacted by dilemma type, in which “personal” dilemmas (e.g., footbridge dilemma) cause greater activations in areas of the brain that are related to affective processing. As a result, there is an instantaneous negative affective response. Contrariwise, “impersonal” dilemmas (e.g., switch dilemma) do not have the conflicting affective response, due to greater activity in areas of the brain that are associated with working memory (utilitarian mode). However, this dual process approach is somewhat artificial [113]. Revisions to this framework by Cushman [112] distinguish between action valuation processes (e.g., shoving the man from the bridge) and outcome valuation processes (e.g., an outcome that causes another severe harm). According to Cushman, the footbridge dilemma involves a complex valuation that assesses and places negative value on actions like pushing another off a bridge. Contrariwise, the switch dilemma represents a simpler model that favors the outcome of saving lives.

Researchers have evolved the classic text-based vignettes of “footbridge” and “trolley” (i.e., switch) dilemmas into virtual footbridge [114] and virtual trolley dilemmas [115,116,117,118]. A benefit of these virtual environment based scenarios is that they have greater ecological validity, and reflect real-world scenarios [22]. This offers an advantage over text-based hypothetical moral dilemmas that may result in gaps in understanding the ways in which the participant response to a paragraph translates into real-world behaviors. As such, virtual environments allow researchers a platform for evaluating morally relevant decision making behaviors in simulated situations. One approach to develop a virtual environment that reflects Greene’s dual process model [111,113] can be found in Navarrete and colleagues’ [115] virtual reality-based switch dilemma. In their study, they compared electrodermal activity of participants as they responded to hypothetical moral decisions in a virtual trolley dilemma. Most participants reported utilitarian outcomes, and increased autonomic arousal was associated with decreased utilitarian endorsements. These findings support a dual process model in which affective activations are related to non-utilitarian moral judgments. These findings were replicated in a study by Skulmowski and colleagues [118], that placed the participant in a first-person (participant drives the train) perspective while immersed in the virtual reality-based trolley dilemma. Findings replicated the behavioral patterns found in text-based studies of the trolley dilemma. Furthermore, they found a peak in the level of arousal related to the moment that the moral decision was made, and context-dependent gaze during the making of a decision to kill. In addition to supporting a dual-process model, this virtual reality platform allowed for decision time frames to be held constant, and events could be logged and labeled for comparison to psychophysiological measurements.

As mentioned above, Cushman [112] revised Greene’s framework to distinguish between action valuation and outcome valuation processes. In another virtual trolley study, Patil et al. [117] found that participants behaved in a utilitarian manner in virtual reality dilemmas, in the face of nonutilitarian judgments, for textual descriptions of the same dilemmas. They contend that moral decision-making in hypothetical moral dilemmas may be impacted by the greater levels of stimulus saliency found in the virtual environment. This comports well with Cushman’s model, in that the saliency of the virtual environment provides a stronger negative value to failures to save threatened victims, than found in the choice to commit a harmful action against one person. Likewise, Francis and colleagues [118] found different behaviors between judgment-based formulations and action-based virtual reality presentations of a footbridge dilemma. These results appear to reflect Cushman’s outcome and action-based valuations. Again, the contextual saliency found in the virtual environment may have resulted in greater negative valuations.

### 6.2. Authority and Obedience in Virtual Environments

Virtual environments have also been used to assess decision making in response to authority. Stanley Milgram’s 1960s study of participants’ willingness to follow instructions and electrically shock a stranger, have been reprised in virtual reality [119]. In a study by Slater and colleagues [119], participants acted as teachers sitting at a desk with an electric shock machine. The participants observed virtual students through a (virtual) partition as they read cue words and administered electric shocks to the virtual student whenever the virtual student responded incorrectly. In concordance with the original Milgram experiment, the virtual student complained and demanded to be released from the experiment. The sample was made up of 34 participants; 23 viewed and heard the virtual student throughout the experiment, and 11 observed and spoke to the virtual student originally, but after a period, a curtain descended and the participants only communicated with the virtual student through text. Results revealed that although participants who communicated by text gave all of the shocks, six of the 23 who observed and heard the virtual student, withdrew from the experiment before giving all shocks. The authors contend that a sense of presence prompts participants to respond to the virtual student as if it were an actual person.

## 7. Virtual Induction of Social Stress

A frequently utilized procedure for social stress induction is the Trier Social Stress Test (TSST) [120]. The TSST requires that the participant develop a five-minute speech that the participant then presents in front of two to three confederates. Furthermore, the participant is instructed to perform a five-minute arithmetic task. The TSST protocol characterizes a representative social evaluative threat that is perceived as outside the participant’s control, because of the confederates’ neutral facial expressions and limited predictability. The TSST has been found to reliably result in significant stress reactions [121,122]. A limitation of the TSST, however, is the requirement that confederates hold their reactions constant for each trial. Given the difficulty in maintaining consistent confederate reactions across studies may limit inter- and intra-individual comparisons [123].

An alternative approach using a virtual reality-based TSST has emerged in a number of studies [123,124,125,126,127,128,129,130,131]. Studies using a virtual reality-based TSST have found significant stress reactions, with peripheral physiological reactions and self-reported stress elevations comparable to those in response to the in vivo TSST. Studies by Kelly and colleagues [127], Ruiz and colleagues [123], and Wållergard and colleagues [131] found that while a virtual reality-based TSST could evoke heart rate responses similar to those found in vivo, cortisol responses were less pronounced in virtual reality-based TSST. In another study, significant blood pressure, heart rate and catecholamine increases were found, but there were no significant changes in plasma cortisol [132].

While these findings are promising, virtual reality-based TSSTs used in these studies have some variance in terms of content. For example, the virtual reality-based TSST used by Fich et al. [125] and Jönsson et al. [126] included three virtual interviewers, whereas Kelly and colleagues [127] used five virtual interviewers. In another study, Delahaye and colleagues [124] ventured far from the three to five interviewers when they used a crowd of 80 virtual characters. Another issue for these studies is that they tended to modify the experimental design by having the virtual audience act as if they were restless, instead of being neutral. Furthermore, while some studies used head mounted displays, Jönsson and colleagues [126] used a room-sized (CAVE-system) virtual reality-based TSST. In Fich and colleagues’ [127] study, the virtual environment was varied to observe behaviors in open and closed spaces.

Given that these modifications may confound the virtual reality-based stress reactions, Kothgassner and colleagues [128] used a virtual reality-based TSST that more closely followed the original TSST protocol. At the start of their virtual reality-based TSST simulation, two virtual interviewers (one male; one female) entered the room, took a seat, and then asked why the human participant was the best candidate for the position. As in the original TSST, participants in the virtual reality-based TSST passed a job-interview in the first half of the task, and then performed an arithmetic task. Importantly, the virtual interviewers maintained neutral expressions throughout the interview. Kothgassner and colleagues [128] used salivary cortisol samples and self-reports as indicators of social stress. Findings revealed a significant increase in cortisol levels and subjective stress. These findings suggest that a virtual reality-based TSST can offer enhanced ecological validity and control for stress research. Moreover, the virtual reality-based TSST may enhance generalization from study findings to real-world settings.

## 8. Virtual Induction of Social Pain

Virtual environments have also been used for studying social exclusion. A notable example is the Cyberball game that has been used for multilevel and experimentally controlled assessments of social exclusion: at the neurobiological level [133], hormonal level [134,135], psychophysiological level [136,137], and the affective processing level [138,139]. The Cyberball game allows for flexible modification of social interactions without the use of live confederates. This is a great asset for studies that aim to collect large samples over the Internet, and for neuroimaging studies. While in many studies participants believe that human participants control the two other avatars with whom the participant is playing catch, telling participants that the avatars in the Cyberball game are controlled by a computer does not change the effects of ostracism [140]. The participant is either included or excluded during the Cyberball tossing game by two of the other avatars, who are controlled by an experimenter. At the start of the Cyberball game, each avatar catches approximately one third of the time and throws a ball approximately one third of the time. For the “inclusion” condition, the participant catches and throws the ball approximately one third of the time. Contrariwise, during the “exclusion” condition, the participant is ostracized as the other two avatars throw the ball back and forth.

Findings have revealed that the experience of being ostracized during the exclusion condition consistently results in increased activation of the dorsal anterior cingulate and anterior insula, which correlates with self-reports of physical pain [133]. Qualitative reviews of neuroimaging findings from Cyberball social exclusion have concluded that nociceptive stimuli and social rejection both activate this physical pain matrix. It is important to note, however, that results from a recent quantitative meta-analysis suggest distinct patterns of activation for nociceptive stimuli and social rejection. Nevertheless, commonalities are still apparent [141]. In another quantitative review, Rotge and colleagues [142] found that ostracism during the Cyberball task activated the dorsal anterior cingulate circuit less than other experimental social pain tasks. These findings support the view that the social pain following ostracism in the Cyberball exclusion condition is less intense than the social pain that from more personal forms of social rejection [133].

Some of this variance may also be accounted for by the fact that early versions of the Cyberball task lacked the everyday realism and ecological validity that are now available in today’s immersive virtual environments. Virtual environment-based Cyberball paradigms have emerged, that place the participant into a virtual environment with interactive virtual humans [128,143,144,145,146]. Findings from these studies reveal that the more immersive virtual environments induced greater feelings of ostracism in participants. Furthermore, the immersive virtual environment Cyberball paradigm allows researchers to control proxemics and non-verbal communications that cannot be achieved in traditional Cyberball presentations. This may enhance flexible manipulation of social information [147].

## 9. Conclusions: Virtual Reality in Social Neuroscience and Beyond

The burgeoning field of social neuroscience offers promise for uncovering the neural underpinnings of social phenomena. Work in this area can be strengthened by adding complex and dynamic stimuli to current approaches that use simple and static stimuli. The study of the neurobiological basis of social cognition requires the development of novel paradigms and methods that are capable of encompassing the inherent complexity of these phenomena. In this review, we have presented a number of assets inherent in virtual reality platforms that may enhance social neuroscience protocols. First, VR offers the social neuroscientist tools for creating realistic simulations of social situations that at same time provide a high degree of experimental control. Second, the social neuroscientist can efficiently administer and integrated virtual reality scenarios into narrative contexts, hence increasing the sense of presence/social presence experienced by the participant. Furthermore, using VR, the social neuroscientist can precisely track the participant’s behavior and synchronize various sources of data to obtain a holistic, integrated measurement of target social interactions. Last, but not least, a unique advantage of VR is that this approach allows social neuroscientists a platform for studying social situations that would otherwise be impossible to investigate using conventional research paradigms (e.g., embodying another self).

### 9.1. Methodological Issues

It is important to note, however, that realizing the potential of VR for social neuroscience requires that several issues be addressed. A first challenge is the integration of immersive VR in functional neuroimaging paradigms. Although progress has been made to allow compatibility between immersive VR technology and neuroimaging systems such as fMRI [148,149], the physical constraints imposed by brain scanners still provide a significant limitation to the experimental options offered by VR. For example, the social scripts that are simulated in VR must be significantly simplified to ensure that stimuli can be repeated enough times to reach statistical power. Furthermore, in VR experiments involving participants’ movements (e.g., in non-verbal communication studies), the integration of fMRI may introduce several constraints employed to avoid experimental artifacts. While these issues are important considerations when designing virtual reality paradigms for fMRI, software is being developed that supports the modeling and usage of VR stimuli. Mueller and colleagues [150] have developed and implemented a VR stimulus application based on C++ that allows for rapid and uncomplicated presentation of VR environments for fMRI studies without any supplementary expert knowledge. Their software allows for the reception of real-time data analytics with a bidirectional communication interface. Furthermore, the internal plugin interface enables social neuroscientists to extend the functionality of the software via custom programmed C++ plugins.

A further issue concerns the complexity associated with the design and execution of VR experiments. Implementing a VR study requires the involvement of varied expertise, which typically includes 3D computer-graphics (for the development of virtual environments), system engineering (for integrating physiological and tracking sensors), artificial intelligence/computer science (for developing computer-based virtual characters) and data science (for the implementation of automatized data analytics). As a consequence, VR-based social neuroscience experiments can easily reach large budgets and may be difficult to replicate (especially for those studies involving the use of large-scale immersive facilities, such as CAVE systems).

Another important limitation of current VR systems for the study of social processes is that most commercially-available immersive VR platforms do not support multiple users, except for a limited number of VR applications (e.g., games). Thus, there is a need for developing VR platforms that are specifically designed for social neuroscience. These platforms should allow the implementation of avatar-based social interaction by tracking (in real time) body movements, facial expressions, and verbal communication. Social neuroscientists can draw from a variety of multiuser platforms found in computer science. For example, Shi and colleagues [151] have developed a multiuser VR system that allows multiple users to interact with others, represented as avatars, in the same virtual environment. Likewise, Noh and Shin [152] have developed a novel connection model for multi-user display and data transmission. Their connection model comprises of application, display, and transmission bindings that cooperate with each other hierarchically. Furthermore, the connection model supports a meta-space that is made up of a configuration control space and a functional control space, which allows for application-specific computing environments that can be implemented at the platform level. Wendrich and colleagues [153] have used hybrid design tool environments to develop social virtual reality scenarios that network commercially available Oculus Rift HMDs.

### 9.2. Ethical Considerations

Progress in social neuroscience is rapidly increasing our knowledge of neural correlates of social cognition. The advent of new technologies (e.g., neuroimaging; virtual environments) and methods raises ethical problems: What are the social and cultural consequences of virtual reality technologies that enable researchers to manipulate the processing of social information? What impact will virtual reality have on our understanding of self and others? There is a need for consensus statements on research parameters and ethical guidelines for VR-based experiments in social neuroscience. The level of realism afforded by current VR systems calls into question the potential psychological effects of exposing participants to extreme social situations that may trigger moral conflicts (such as those implicated by experiments that involve the reproduction of ethical dilemmas). An example is the virtual reprise of Milgram’s experiments, in which participants were instructed to administer “electric shocks” to computer-generated characters [154]. To understand the specific ethical boundaries of VR-based experiments, Madary and Metzinger [155] recommend that researchers “follow the principle of non-maleficence: do no harm… In its general form, the principle of non-maleficence for VR can be expressed as follows: No experiment should be conducted using virtual reality with the foreseeable consequence that it will cause serious or lasting harm to a subject”. In summary, virtual reality research should be performed in a beneficent research environment, with the aim of mitigating risks for users of virtual reality. Given the importance of these issues, continuous reflection and discussion of these issues is needed (see Bush and Schatz [156] for a recent discussion of ethical and methodological considerations for using advanced technologies).

Virtual reality-based social neuroscience scenarios offer promise for emotionally engaging background narratives to enhance affective experience and social interactions. It is important to emphasize that the use of virtual environments advocated here does not seek to minimize the contribution of traditional (static pictures; vignettes; scripted imagery of social events) stimuli found in social neuroscience. The static stimuli in social neuroscience have numerous benefits for researchers, as evidenced by the progress that has been made using such tests and stimuli. The emphasis herein is upon extending social neuroscience paradigms via dynamic virtual environments for investigating real-world social questions.

## References

[1] Cacioppo J.T., Berntson G.G., Decety J. (2010). Social neuroscience and its relation to social psychology. Soc. Cogn..

[2] Cacioppo J.T., Berntson G.G. (1992). Social psychological contributions to the decade of the brain: Doctrine of multilevel analysis. Am. Psychol..

[3] Ochsner K.N., Lieberman M.D. (2001). The emergence of social cognitive neuroscience. Am. Psychol..

[4] Moscowitz G.B. (2005). Social Cognition: Understanding Self and Others.

[5] Bradley M.M., Lang P.J., Coan J.A., Allen J.J.B. (2007). The International Affective Picture System (IAPS) in the Study of emotion and attention. Handbook of Emotion Elicitation and Assessment.

[6] Fusar-Poli P., Placentino A., Carletti F., Landi P., Allen P., Surguladze S., Benedetti F., Abbamonte M., Gasparotti R., Barale F. (2009). Functional atlas of emotional faces processing: A voxel-basedmeta-analysis of 105 functional magnetic resonance imaging studies. J. Psychiatry Neurosci..

[7] Burnett S., Bird G., Moll J., Frith C., Blakemore S.J. (2009). Development during adolescence of the neural processing of social emotion. J. Cogn. Neurosci..

[8] Dong S.Y., Kim B.K., Lee S.Y. (2016). Implicit agreeing/disagreeing intention while reading self relevant sentences: A human fMRI study. Soc. Neurosci..

[9] Bartholow B.D., Fabiani M., Gratton G., Bettencourt B.A. (2001). A psychophysiological examination of cognitive processing of and affective responses to social expectancy violations. Psychol. Sci..

[10] Jerónimo R., Volpert H.I., Bartholow B.D. (2017). Event-related potentials reveal early attention bias for negative, unexpected behavior. Soc. Neurosci..

[11] Frewen P.A., Dozois D.J.A., Neufeld R.W.J., Densmore M., Stevens T.K., Lanius R.A. (2011). Neuroimaging social emotional processing in women: fMRI study ofscript-driven imagery. Soc. Cogn. Affect. Neurosci..

[12] Chiu I., Gfrorer R.I., Piguet O., Berres M., Monsch A.U., Sollberger M. (2015). “Now I see it, now I don’t”: Determining Threshold Levels of Facial Emotion Recognition for Use in Patient Populations. J. Int. Neuropsychol. Soc..

[13] Henry J.D., Cowan D.G., Lee T., Sachdev P.S. (2015). Recent trends in testing social cognition. Curr. Opin. Psychiatry.

[14] Zaki J., Ochsner K. (2009). The need for a cognitive neuroscience of naturalistic social cognition. Ann. N. Y. Acad. Sci..

[15] Risko E.F., Laidlaw K.E., Freeth M., Foulsham T., Kingstone A. (2012). Social attention with real versus reel stimuli: Toward an empirical approach to concerns about ecological validity. Front. Hum. Neurosci..

[16] Chakrabarti B. (2013). Parameterising ecological validity and integrating individual differences within second-person neuroscience. Behav. Brain Sci..

[17] Ochsner K.N. (2004). Current directions in social cognitive neuroscience. Curr. Opin. Neurobiol..

[18] Schilbach L., Wohlschläger A.M., Newen A., Krämer N., Shah N.J., Fink G.R., Vogeley K. (2006). Being with others: Neural correlates of social interaction. Neuropsychologia.

[19] Schilbach L., Timmermans B., Reddy V., Costall A., Bente G., Schlicht T., Vogeley K. (2013). Toward a second-person neuroscience. Behav. Brain Sci..

[20] Schilbach L. (2015). Eye to eye, face to face and brain to brain: Novel approaches to study the behavioral dynamics and neural mechanisms of social interactions. Curr. Opin. Behav. Sci..

[21] Bohil C.J., Alicea B., Biocca F.A. (2011). Virtual reality in neuroscience research and therapy. Nat. Rev. Neurosci..

[22] Parsons T.D. (2015). Virtual Reality for Enhanced Ecological Validity and Experimental Control in the Clinical, Affective, and Social Neurosciences. Front. Hum. Neurosci..

[23] Gorini A., Capideville C.S., DeLeo G., Mantovani F., Riva G. (2011). The role of immersion and narrative in mediated presence: The virtual hospital experience. Cyberpsychol. Behav. Soc. Netw..

[24] Diemer J.E., Alpers G.W., Peperkorn H.M., Shiban Y., Mühlberger A. (2015). The impact of perception and presence on emotional reactions: A review of research in virtual reality. Front. Psychol..

[25] Bailenson J.N., Blascovich J. (2004). Avatars. Encyclopedia of Human-Computer Interaction.

[26] Kenny P., Parsons T.D., Perez-Marin C., Pascual-Nieto I. (2011). Embodied conversational virtual human patients. Conversational Agents and Natural Language Interaction:Techniques and Effective Practices.

[27] Bowman D., Kruijff E., LaViola J., Poupyrev I. (2001). An introduction to 3-D user interface design. Presence Teleoper. Virtual Environ..

[28] Fuchs P., Guitton P., Fuchs P., Morequ G., Guitton P. (2011). Introduction to virtual reality. Virtual Reality: Concepts And Technologies.

[29] Cruz-Neira C., Sandin D.J., DeFanti T.A., Kenyon R.V., Hart J.C. (1992). The CAVE. Audio visual experience automatic virtual environment. Commun. ACM.

[30] Slater M., Wilbur S. (1997). A framework for immersive virtual environments (FIVE): Speculations on the role of presence in virtual environments. Presence Teleoper. Virtual Environ..

[31] Sheridan T.B. (1992). Musings on Telepresence and Virtual Presence. Presence Teleoper. Virtual Environ..

[32] IJsselsteijn W.A., de Ridder H., Freeman J., Avons S.E., Bouwhuis D.G. (2001). Effects of stereoscopic presentation, image motion, and screen size on subjective and objective corroborative measures of presence. Presence Teleoper. Virtual Environ..

[33] Parsons T.D., Barnett M., Melugin P. (2015). Assessment of Personality and Absorption for Mediated Environments in a College Sample. Cyberpsychol. Behav. Soc. Netw..

[34] Sacau A., Laarni J., Hartmann T. (2008). Influence of individual factors on presence. Comput. Hum. Behav..

[35] Zahorik P., Jenison R.L. (1998). Presence as being-in-the-world. Presence Teleoper. Virtual Environ..

[36] Gibson J.J., Shaw R., Bransford J. (1977). The theory of affordances. Perceiving, Acting, and Knowing.

[37] Short J.A., Williams E., Christie B. (1976). The Social Psychology of Telecommunications.

[38] Sanchez-Vives M.V., Slater M. (2005). From presence to consciousness through virtual reality. Nat. Rev. Neurosci..

[39] Macedonio M.F., Parsons T.D., Digiuseppe R.A., Weiderhold B.A., Rizzo A.A. (2007). Immersiveness and physiological arousal within panoramic video-based virtual reality. Cyberpsychol. Behav..

[40] Baumgartner T., Valko L., Esslen M., Jäncke L. (2006). Neural correlate of spatial presence in an arousing and noninteractive virtual reality: An EEG and psychophysiology study. CyberPsychol. Behav..

[41] Baumgartner T., Speck D., Wettstein D., Masnari O., Beeli G., Jäncke L. (2008). Feeling present in arousing virtual reality worlds: Prefrontal brain regions differentially orchestrate presence experience in adults and children. Front. Hum. Neurosci..

[42] Alcañiz M., Rey B., Tembl J., Parkhutik V. (2009). A Neuroscience Approach to Virtual Reality Experience Using Transcranial Doppler Monitoring. Presence Teleoper. Virtual Environ..

[43] Lombard M., Biocca F., Freeman J., IJsselsteijn W., Schaevitz R.J. (2015). Immersed in Media. Telepresence Theory, Measurement & Technology.

[44] Riva G., Waterworth J.A., Murray D. (2014). Interacting with Presence: HCI and the Sense of Presence in Computer-Mediated Environments.

[45] Lombard M., Ditton T. (1997). At the heart of it all: The concept of presence. J. Comput. Mediat. Commun..

[46] Waterworth J.A., Riva G. (2014). Feeling Present in the Physical World and in Computer-Mediated Environments.

[47] Biocca F. (1997). The Cyborg’s Dilemma: Progressive Embodiment in Virtual Environments. J. Comput. Mediat. Commun..

[48] Jenkinson P.M., Preston C. (2015). New reflections on agency and body ownership: The moving rubber hand illusion in the mirror. Conscious. Cogn..

[49] Tsakiris M., Schutz-Bosbach S., Gallagher S. (2007). On agency and body-ownership: Phenomenological and neurocognitive reflections. Conscious. Cogn..

[50] Biocca F., Harms C., Burgoon J.K. (2003). Toward a more robust theory and measure of social presence: Review and suggested criteria. Presence Teleoper. Virtual Environ..

[51] Riva G., Mantovani F. (2014). Extending the self through the tools and the others: A general framework for presence and social presence in mediated interactions. Interacting with Presence: HCI and the Sense of Presence in Computer-mediated Environments.

[52] Wallwork S.B., Bellan V., Catley M.J., Moseley G.L. (2016). Neural representations and the corticalbody matrix: Implications for sports medicine and future directions. Br. J. Sports Med..

[53] Riva G. (2008). Enacting interactivity: The role of presence. Emerg. Commun..

[54] Riva G. (2009). Is presence a technology issue? Some insights from cognitive sciences. Virtual Real..

[55] Riva G., Waterworth J.A., Grimshaw M. (2014). Being present in a virtual world. The Oxford Handbook of Virtuality.

[56] Riva G., Waterworth J.A., Waterworth E.L., Mantovani F. (2011). From intention to action: The role of presence. New Ideas Psychol..

[57] Russell J.A. (1996). Agency: Its Role in Mental Development.

[58] Friston K. (2010). The free-energy principle: A unified brain theory?. Nat. Rev. Neurosci..

[59] Friston K., Daunizeau J., Kilner J., Kiebel S.J. (2010). Action and behavior: A free-energy formulation. Biol. Cybern..

[60] Knoblich G., Thornton I., Grosjean M., Shiffrar M. (2005). Human Body Perception from the Inside Out.

[61] Wilson M., Knoblich G. (2005). The case for motor involvement in perceiving conspecifics. Psychol. Bull..

[62] Rizzolatti G., Fadiga L., Gallese V., Fogassi L. (1996). Premotor cortex and the recognition of motor actions. Cogn. Brain Res..

[63] Rizzolatti G., Sinigaglia C. (2006). Milano. So quel che fai. Il Cervello Che Agisce e i Neuroni Specchio.

[64] Knoblich G., Flach R. (2003). Action identity: Evidence from self-recognition, prediction, and coordination. Conscious. Cogn..

[65] Prinz W. (1997). Perception and action planning. Eur. J. Cogn. Psychol..

[66] Becchio C., Bertone C. (2005). Il paradosso dell’intenzionalità collettiva. G. Ital. Psicol..

[67] Searle J. (1983). Intentionality: An Essay in the Philosophy of Mind.

[68] Pacherie E. (2008). The phenomenology of action: A conceptual framework. Cognition.

[69] Riva G., Mantovani F., Waterworth E.L., Waterworth J.A. (2015). Intention, action, self and other: An evolutionary model of presence. Immersed in Media.

[70] Riva G., Mantovani F. (2012). From the body to the tools and back: A general framework for presence in mediated interactions. Interact. Comput..

[71] Searle J., Cohen P., Morgan J., Pollack M.E. (1990). Collective Intentions and Actions. Intentions in Communication.

[72] Reader A.T., Holmes N.P. (2016). Examining ecological validity in social interaction: Problems of visual fidelity, gaze, and social potential. Cult. Brain.

[73] de Jaegher H., di Paolo E., Gallagher S. (2010). Can social interaction constitute social cognition?. Trends Cogn. Sci..

[74] Gregory N.J., López B., Graham G., Marshman P., Bate S., Kargas N. (2015). Reduced gaze following and attention to heads when viewing a “live” social scene. PLoS ONE.

[75] Hogenelst K., Schoevers R.A., aan het Rot M. (2015). Studying the neurobiology of human social interaction: Making the case for ecological validity. Soc. Neurosci..

[76] Parsons T.D. (2017). Cyberpsychology and the Brain: The Interaction of Neuroscience and Affective Computing.

[77] Bombari D., Schmid Mast M., Cañadas E., Bachmann M. (2015). Studying social interactions through immersive virtual environment technology: Virtues, pitfalls, and future challenges. Front. Psychol..

[78] Georgescu A.L., Kuzmanovic B., Roth D., Bente G., Vogeley K. (2014). The Use of Virtual Characters to Assess and Train Non-Verbal Communication in High-Functioning Autism. Front. Hum. Neurosci..

[79] Virtual Reality Peripheral Network - Official Repo. https://github.com/vrpn/vrpn/wiki.

[80] Steptoe W., Steed A. (2012). Multimodal Data Capture and Analysis of Interaction in Immersive Collaborative Virtual Environments. Presence Teleoper. Virtual Environ..

[81] Bente G., Krämer N.C., Eschenburg F., Konijn E.A., Utz S., Tanis M., Barnes S.B. (2008). Is there anybody out there? Analyzing the effects of embodiment and nonverbal behavior in avatar-mediated communication. Mediated Interpersonal Communication.

[82] Bente G., Krämer N.C., Kappas A., Krämer N.C. (2011). Virtual gestures: Embodiment and nonverbal behavior in computer-mediated communication. Face-To-Face Communication Over the Internet: Issues, Research, Challenges.

[83] Bailenson J.N., Beall A.C., Loomis J., Blascovich J., Turk M. (2004). Transformed Social Interaction: Decoupling Representation from Behavior and Form in Collaborative Virtual Environments. Presence Teleoper. Virtual Environ..

[84] Bailenson J.N., Beall A.C., Blascovich J., Loomis J., Turk M. (2005). Transformed Social Interaction, Augmented Gaze, and Social Influence in Immersive Virtual Environments. Hum. Commun. Res..

[85] Borst A.W., Gelder B.D. (2015). Is it the real deal? Perception of virtual characters versus humans: An affective cognitive neuroscience perspective. Front. Psychol..

[86] Dyck M., Winbeck M., Leiberg S., Chen Y., Gur R.C., Mathiak K. (2008). Recognition profile of emotions in natural and virtual faces. PLoS ONE.

[87] McDonnell R., Jorg S., McHugh J., Newell F., O’Cullivan C. (2008). Evaluating the emotional content of human motions on real and virtual characters. Proceedings of the 5th Symposium on Applied Perception in Graphics and Visualization APGV.

[88] Moser E., Derntl B., Robinson S., Fink B., Gur R.C., Grammer K. (2007). Amygdala activation at 3T in response to human and avatar facial expressions of emotions. J. Neurosci. Methods.

[89] Friedman D., Pizarro R., Or-Berkers K., Neyret S., Pan X., Slater M. (2014). A method for generating an illusion of backwards time travel using immersive virtual reality-an exploratory study. Front. Psychol..

[90] Slater M., Sanchez-Vives M.V. (2014). Transcending the self in immersive virtual reality. Computer.

[91] Chirico A., Gaggioli A., Riva G., Yaden D.B. (2016). The Potential of Virtual Reality for the Investigation of Awe. Front. Psychol..

[92] Serino S., Pedroli E., Keizer A., Triberti S., Dakanalis A., Pallavicini F., Chirico A., Riva G. (2016). Virtual reality body swapping: A tool for modifying the allocentric memory of the body. Cyberpsychol. Behav. Soc. Netw..

[93] Yee N., Bailenson J. (2007). The Proteus Effect: The Effect of Transformed Self-Representation on Behavior. Hum. Commun. Res..

[94] Yee N., Bailenson J.N., Ducheneaut N. (2009). The Proteus effect: Implications of transformed digital self-representation on online and offline behavior. Commun. Res..

[95] Dotsch R., Wigboldus D.H.J. (2008). Virtual prejudice. J. Exp. Soc. Psychol..

[96] Parsons T.D., Kenny P., Cosand L., Iyer A., Courtney C., Rizzo A.A. (2009). A Virtual Human Agent for Assessing Bias in Novice Therapists. Stud. Health Technol. Inform..

[97] Maister L., Sebanz N., Knoblich G., Tsakiris M. (2013). Experiencing ownership over a dark-skinned body reduces implicit racial bias. Cognition.

[98] Peck T.C., Seinfeld S., Aglioti S.M., Slater M. (2013). Putting yourself in the skin of a black avatar reduces implicit racial bias. Conscious. Cogn..

[99] Slater M., Spanlang B., Sanchez-Vives M.V., Blanke O. (2010). First person experience of body transfer in virtual reality. PLoS ONE.

[100] Banakou D., Groten R., Slater M. (2013). Illusory ownership of a virtual child body causes overestimation of object sizes and implicit attitude changes. Proc. Natl. Acad. Sci. USA.

[101] Van der Hoort B., Guterstam A., Ehrsson H.H. (2011). Being barbie: The size of one’s own bodydetermines the perceived size of the world. PLoS ONE.

[102] Preston C., Ehrsson H.H. (2014). Illusory changes in body size modulate body satisfaction in a way that is related to non-clinical eating disorder psychopathology. PLoS ONE.

[103] Normand J.M., Giannopoulos E., Spanlang B., Slater M. (2011). Multisensory stimulation can induce an illusion of larger belly size in immersive virtual reality. PLoS ONE.

[104] Carter E.J., Pelphrey K.A. (2008). Friend or foe? Brain systems involved in the perception of dynamic signals of menacing and friendly social approaches. Soc. Neurosci..

[105] Caruana N., Brock J., Woolgar A. (2015). A frontotemporoparietal networkcommon to initiating and responding to joint attention bids. Neuroimage.

[106] Oberwelland E., Schilbach L., Barisic I., Krall S.C., Vogeley K., Fink G.R., Herpertz-Dahlmann B., Konrad K., Schulte-Rüther M. (2016). Look into my eyes: Investigating joint attention using interactive eye-tracking and fMRI in a developmental sample. NeuroImage.

[107] Redcay E., Kleiner M., Saxe R. (2012). Look at this: The neural correlates of initiating and responding to bids for joint attention. Front. Hum. Neurosci..

[108] Schilbach L., Wilms M., Eickhoff S.B., Romanzetti S., Tepest R., Bente G., Shah N.J., Fink G.R., Vogeley K. (2010). Minds made for sharing: Initiating jointattention recruits reward-related neurocircuitry. J. Cogn. Neurosci..

[109] Wilms M., Schilbach L., Pfeiffer U., Bente G., Fink G.R., Vogeley K. (2010). It’s in your eyes? using gaze-contingent stimuli to create truly interactive paradigmsfor social cognitive and affective neuroscience. Soc. Cogn. Affect. Neurosci..

[110] Foot P. (1978). Virtues and Vices and Other Essays in Moral Philosophy.

[111] Greene J.D., Sommerville R.B., Nystrom L.E., Darley J.M., Cohen J.D. (2001). An fMRI investigation of emotional engagement in moral judgment. Science.

[112] Cushman F. (2013). Action, outcome, and value a dual-system framework for morality. Pers. Soc. Psychol. Rev..

[113] Greene J.D., Nystrom L.E., Engell A.D., Darley J.M., Cohen J.D. (2004). The neural bases of cognitive conflict and control in moral judgment. Neuron.

[114] Francis K.B., Howard C., Howard I.S., Gummerum M., Ganis G., Anderson G., Terbeck S. (2016). Virtual Morality: Transitioning from Moral Judgment to Moral Action?. PLoS ONE.

[115] Navarrete C.D., McDonald M.M., Mott M.L., Asher B. (2012). Virtual morality: Emotion and action in a simulated three-dimensional “trolley problem”. Emotion.

[116] Pan X., Slater M. (2011). Confronting a moral dilemma in virtual reality: A pilot study. Proceedings of the 25th BCS Conference on Human-Computer Interaction.

[117] Patil I., Cogoni C., Zangrando N., Chittaro L., Silani G. (2014). Affective basis of judgment-behavior discrepancy in virtual experiences of moral dilemmas. Soc. Neurosci..

[118] Skulmowski A., Bunge A., Kaspar K., Pipa G. (2014). Forced-choice decision-making in modified trolley dilemma situations: A virtual reality and eye tracking study. Front. Behav. Neurosci..

[119] Slater M., Antley A., Davison A., Swapp D., Guger C., Barker C., Pistrang N., Sanchez-Vives M.V. (2006). A virtual reprise of the Stanley Milgram obedience experiments. PLoS ONE.

[120] Kirschbaum C., Pirke K.M., Hellhammer D.H. (1993). The ‘Trier Social Stress Test’—A tool for investigating psychobiological stress responses in a laboratory setting. Neuropsychobiology.

[121] Kudielka B.M., Buske-Kirschbaum A., Hellhammer D.H., Kirschbaum C. (2004). HPA axis responses to laboratory psychosocial stress in healthy elderly adults, younger adults, and children: Impact of age and gender. Psychoneuroendocrinology.

[122] Kudielka B.M., Schommer N.C., Hellhammer D.H., Kirschbaum C. (2004). Acute HPA axis responses, heart rate, and mood changes to psychosocial stress (TSST) in humans at different times of day. Psychoneuroendocrinology.

[123] Ruiz A.S., Peralta-Ramirez M.I., Garcia-Rios M.C., Muñoz M.A., Navarrete-Navarrete N., Blazquez-Ortiz A. (2010). Adaptation of the trier social stress test to virtual reality: Psycho-physiological and neuroendocrine modulation. J. Cyber Ther. Rehabil..

[124] Delahaye M., Lemoine P., Cartwright S., Deuring G., Beck J., Pflueger M., Hachtel H. (2015). Learning aptitude, spatial orientation and cognitive flexibility tested in a virtual labyrinth after virtual stress induction. BMC Psychol..

[125] Fich L.B., Jönsson P., Kirkegaard P.H., Wallergård M., Garde A.H., Hansen Å. (2014). Can architectural design alter the physiological reaction to psychosocial stress? A virtual TSST experiment. Physiol. Behav..

[126] Jönsson P., Wallergård M., Österberg K., Hansen Å.M., Johansson G., Karlson B. (2010). Cardiovascular and cortisol reactivity and habituation to a virtual reality version of the Trier Social Stress Test: A pilot study. Psychoneuroendocrinology.

[127] Kelly O., Matheson K., Martinez A., Merali Z., Anisman H. (2007). Psychosocial stress evoked by a virtual audience: Relation to neuroendocrine activity. Cyberpsychol. Behav..

[128] Kothgassner O.D., Hlavacs H., Beutl L., Glenk L.M., Palme R., Felnhofer A. (2016). Two experimental virtual paradigms for stress research: Developing avatar-based approaches for interpersonal and evaluative stressors. Proceedings of the International Conference on Entertainment Computing ICEC 2016.

[129] Montero-López E., Santos-Ruiz A., García-Ríos M.C., Rodríguez-Blázquez R., Pérez-García M., Peralta-Ramírez M.I. (2016). A virtual reality approach to the Trier Social Stress Test: Contrasting two distinct protocols. Behav. Res. Methods.

[130] Shiban Y., Diemer J., Brandl S., Zack R., Mühlberger A., Wüst S. (2016). Trier social stress test in vivo and in virtual reality: Dissociation of response domains. Int. J. Psychophysiol..

[131] Wallergård M., Jönsson P., Johansson G., Karlson B. (2011). A virtual reality version of the Trier Social Stress Test: A pilot study. Presence.

[132] Kotlyar M., Donahue C., Thuras P., Kushner M.G., O’Gorman N., Smith E.A., Adson D.E. (2008). Physiological response to a speech stressor presented in a virtual reality environment. Psychophysiology.

[133] Eisenberger N.I. (2012). The neural bases of social pain: Evidence for shared representations with physical pain. Psychosom. Med..

[134] Geniole S.N., Carré J.M., McCormick C.M. (2011). State, not trait, neuroendocrine function predicts costly reactive aggression in men after social exclusion and inclusion. Biol. Psychol..

[135] Zwolinski J. (2012). Psychological and neuroendocrine reactivity to ostracism. Aggress. Behav..

[136] Moor B.G., Crone E.A., van der Molen M.W. (2010). The heartbrake of social rejection heart rate deceleration in response to unexpected peer rejection. Psychol. Sci..

[137] Sijtsema J.J., Shoulberg E.K., Murray-Close D. (2011). Physiological reactivity and different forms of aggression in girls: Moderating roles of rejection sensitivity and peer rejection. Biol. Psychol..

[138] Wesselmann E.D., Wirth J.H., Mroczek D.K., Williams K.D. (2012). Dial a feeling: Detecting moderation of affect decline during ostracism. Pers. Individ. Differ..

[139] Williams K.D. (2007). Ostracism. Annu. Rev. Psychol..

[140] Zadro L., Williams K.D., Richardson R. (2004). How low can you go? Ostracism by a computer lowers belonging, control, self-esteem and meaningful existence. J. Exp. Soc. Psychol..

[141] Cacioppo S., Frum C., Asp E., Weiss R.M., Lewis J.W., Cacioppo J.T. (2013). A quantitative meta-analysis of functional imaging studies of social rejection. Sci. Rep..

[142] Rotge J.Y., Lemogne C., Hinfray S., Huguet P., Grynszpan O., Tartour E., George N., Fossati P. (2014). A meta analysis of the anterior cingulate contribution to social pain. Soc. Cogn. Affect. Neurosci..

[143] Kassner M.P., Wesselmann E.D., Law A.T., Williams K.D. (2012). Virtually ostracized: Studying ostracism in immersive virtual environments. Cyberpsychol. Behav. Soc. Netw..

[144] Kothgassner O.D., Kafka J., Rudyk J., Beutl L., Hlavacs H., Felnhofer A. Does Social Exclusion Hurt Virtually Like It Hurts in Real Life? the Role of Agency and Social Presence in the Perception and Experience of Social Exclusion. https://pdfs.semanticscholar.org/34b8/5e309ee0732ba0d4c4c61d33f3f8788e06b9.pdf.

[145] Segovia K.Y., Bailenson J.N. (2012). Virtual imposters: Responses to avatars that do not look like their controllers. Soc. Influ..

[146] Venturini E., Riva P., Serpetti F., Romero L., Pallavincini F., Mantovani F., Cloutier R., McMahan T., Stonecipher K., Parsons T.D. (2016). A comparison of 3D versus 2D virtual environments on the feelings of social exclusion, inclusion and over-inclusion. Annu. Rev. Cyber Ther. Telemed..

[147] Wirth J.H., Sacco D.F., Hugenberg K., Williams K.D. (2010). Eye gaze as relational evaluation: Averted eye gaze leads to feelings of ostracism and relational devaluation. Pers. Soc. Psychol. Bull..

[148] Adamovich S.V., August K., Merians A., Tunik E. (2009). A virtual reality-based system integrated with fmri to study neural mechanisms of action observation-execution: A proof of concept study. Restor. Neurol. Neurosci..

[149] Baumann S., Neff C., Fetzic S., Stangl G., Basler L., Vereneck R., Schneider W. (2003). A virtual reality system for neurobehavioral and functional MRI studies. Cyberpsychol. Behav..

[150] Mueller C., Luehrs M., Baecke S., Adolf D., Luetzkendorf R., Luchtmann M., Bernarding J. (2012). Building virtual reality fMRI paradigms: A framework for presenting immersive virtual environments. J. Neurosci. Methods.

[151] Shi Y., Du J., Lavy S., Zhao D. (2016). A Multiuser Shared Virtual Environment for Facility Management. Procedia Eng..

[152] Noh W.J., Shin C. (2016). Novel Control Framework for Multi-User Display and Data Transmission in Networked Virtual Reality Services. J. Nanoelectron. Optoelectron..

[153] Wendrich R.E., Chambers K.-H., Al-Halabi W., Seibel E.J., Grevenstuk O., Ullman D., Hoffman H.G. Hybrid Design Tools in a Social Virtual Reality Using Networked Oculus Rift: A Feasibility Study in Remote Real-Time Interaction. Proceedings of the 36th Computers and Information in Engineering Conference.

[154] Antley A., Barker C., Davison A., Guger C., Pistrang N., Slater M., Swapp D., Sanchez Vives M.V. (2006). A Virtual Reprise of the Stanley Milgram Obedience Experiments. PLoS ONE.

[155] Madary M., Metzinger T. (2016). Real Virtuality: A Code of Ethical Conduct. Recommendations for Good Scientific Practice and the Consumers of VR-Technology. Front. Robot. AI.

[156] Bush S.S., Schatz P., Kane R., Parsons T.D. (2017). Advanced technology and assessment: Ethical and methodological considerations. The Role of Technology in Clinical Neuropsychology.

